# Mitochondria directly donate their membrane to form autophagosomes during a novel mechanism of parkin-associated mitophagy

**DOI:** 10.1186/2045-3701-4-16

**Published:** 2014-03-27

**Authors:** Katherine L Cook, David R Soto-Pantoja, Mones Abu-Asab, Pamela AG Clarke, David D Roberts, Robert Clarke

**Affiliations:** 1 Department of Oncology and Lombardi Comprehensive Cancer Center, Georgetown University Medical Center, Washington, DC 20057, USA; 2 Laboratory of Pathology, Center for Cancer Research, National Cancer Institute, National Institutes of Health, Bethesda, MD 20892, USA; 3 Immunopathology Section, Laboratory of Immunology, National Eye Institute National Institutes of Health, Bethesda, MD 20892, USA

**Keywords:** Breast cancer, Mitochondria, Autophagy, Mitophagy, Parkin, Antiestrogen resistance, Fulvestrant, Imatinib, Estrogen receptor-α

## Abstract

**Background:**

Autophagy (macroautophagy), a cellular process of “self-eating”, segregates damaged/aged organelles into vesicles, fuses with lysosomes, and enables recycling of the digested materials. The precise origin(s) of the autophagosome membrane is unclear and remains a critical but unanswered question. Endoplasmic reticulum, mitochondria, Golgi complex, and the plasma membrane have been proposed as the source of autophagosomal membranes.

**Findings:**

Using electron microscopy, immunogold labeling techniques, confocal microscopy, and flow cytometry we show that mitochondria can directly donate their membrane material to form autophagosomes. We expand upon earlier studies to show that mitochondria donate their membranes to form autophagosomes during basal and drug-induced autophagy. Moreover, electron microscopy and immunogold labeling studies show the first physical evidence of mitochondria forming continuous structures with LC3-labeled autophagosomes. The mitochondria forming these structures also stain positive for parkin, indicating that these mitochondrial-formed autophagosomes represent a novel mechanism of parkin-associated mitophagy.

**Conclusions:**

With the on-going debate regarding autophagosomal membrane origin, this report demonstrates that mitochondria can donate membrane materials to form autophagosomes. These structures may also represent a novel form of mitophagy where the mitochondria contribute to the formation of autophagosomes. This novel form of parkin-associated mitophagy may be a more efficient bio-energetic process compared with *de novo* biosynthesis of a new membrane, particularly if the membrane is obtained, at least partly, from the organelle being targeted for later degradation in the mature autolysosome.

## Findings

Autophagy involves the segregation of subcellular material into double membrane structures (autophagosomes) that then fuse with lysosomes (autolysosomes) wherein the cellular cargo is subsequently degraded by lysosomal hydrolases. This process facilitates the digestive degradation of aged, damaged, or unneeded organelles including mitochondria, Golgi complex, and endoplasmic reticulum [1]. Understanding of the autophagic machinery has advanced; however the primary source of the phospholipid bilayer that creates the autophagosome membrane has remained unclear [2,3].

The difficulty in identifying the origin of cellular material donated to form autophagosome membranes reflects the inability of specific markers for each subcellular organelle to carry over to autophagosomes. Thus, various organelles have been proposed to be autophagosome membrane donors including the plasma membrane, endoplasmic reticulum, Golgi complex, mitochondria, and even a *de novo* generation model [2,3]. The endoplasmic reticulum was originally implicated by studies reporting the concurrent presence of rough endoplasmic reticulum integral membrane proteins both in autophagosome membrane preparations and electron microscopy images [4,5]. However, contradictory data emerged indicating only 30% of all autophagosomes are associated with the endoplasmic reticulum, suggesting the involvement of other organelles in the formation of autophagosomes [6]. More recently, the outer mitochondrial membrane was proposed to serve as a donor source for starvation-induced autophagosome formation [7]. Time-lapse photography data suggested that the early autophagy protein *ATG5* and the autophagosomal marker LC3 translocate to puncta localized on mitochondria, and that labeled outer mitochondrial membrane protein concurrently marked both autophagosomes and mitochondria in data obtained following serum starvation of a rat kidney cell line [8,9]. However, this study is limited because of the primary use of confocal microscopy and the general observation that localization is to be anticipated since the mitochondria are engulfed within mature autophagosomes during mitophagy. The resolution provided by electron microscopy (EM) is needed to directly show autophagosome structures, their content, and their special relationships with mitochondria; this evidence has been notably lacking. We show, for the first time, visual evidence of the contribution of mitochondrial membrane donation to autophagosome formation in both basal and drug-induced autophagy in a human breast cancer cell line. Moreover, these mitochondria donating membranes to form autophagosomes stain positive for the mitophagy-related protein parkin, suggesting a novel mechanism of mitophagy whereby the mitochondria contribute to autophagosome formation, other than being engulfed by the forming autophagosome [10].

## Materials and methods

The following materials were obtained as indicated: Imatinib and ICI 182,780 (Tocris Bioscience, Ellisville, MO); penicillin and Improved Minimal Essential Medium (IMEM; Gibco Invitrogen BRL, Carlsbad, CA); bovine calf charcoal stripped serum (CCS) (Equitech-Bio Inc, Kerrville, TX); Lipofectamine RNAiMax reagent (Invitrogen); Estrogen receptor-α (ER) shRNA (Evrogen, Moscow, Russia); GFP-LC3 (Addgene, Cambridge, MA); EndoTracker Red, Golgi-RFP, MitoTracker-GFP, MitoTracker-RFP (Invitrogen); Cyto-ID Autophagosome detection kit (Enzo Life Sciences); LC3B and parkin antibody (Cell Signaling Technology, Danvers, MA); PINK1 and parkin siRNA (Origene, Rockville, MD).

LCC9 breast carcinoma cells were grown in phenol-red free IMEM media containing 5% CCS. Cells were grown at 37°C in a humidified, 5% CO_2_:95% air atmosphere. Cells were plated in 10 cm dishes and treated with 0.1% v/v ethanol vehicle, 100 nM fulvestrant, or 10 μM Imatinib for 72 hours, or transfected with four shRNA constructs targeting ER-α. EM was performed as previously described [11]. Briefly, cells were pelleted and fixed with 2.5% glutaraldehyde and postfixed with 0.5% osmium tetroxide. Cells were then dehydrated and embedded in Spurs epoxy resin. Embedded cells were cut into ultrathin sections (90 nm), double-stained with uranyl acetate and lead citrate, and viewed with a Philips CM10 transmission electron microscope (Phillips Electronics). Autophagosome number and size were quantified using ImageJ software.

LCC9 cells were transfected with GFP-LC3B and control or ERα shRNA, 0.1% v/v ethanol vehicle, 500 nM ICI, or 10 μM Imatinib and with lentiviral RFP-labeled organelle trackers (endoplasmic reticulum, golgi complex, and mitochondria) for 24 hours. Cells were counterstained with DAPI and confocal microscopy was performed using an Olympus IX-70 confocal microscope (LCCC Imaging Shared Resources) to determine LC3-positive punctate formation and LC3 co-localization with different cellular organelles. LCC9 cells were treated with vehicle, serum starvation, 500 nM ICI, 2 ng/mL tunicamycin, transfected with ATG7 siRNA (negative control), transfected with ERα shRNA, transfected with parkin siRNA, or treated with 10 μM Imatinib for 48 hours. Cells were incubated with MitoTracker-GFP for 24 hours prior to cell harvesting. Cells were collected and treated with a modified monodansylcadaverine. Cells were sorted by flow cytometry to quantify autophagosome and mitochondria number (LCCC Flow Cytometry Shared Resource).

The effect of mitophagy on antiestrogen responsiveness was determined by crystal violet cell density assay. Briefly, 5 x 10^3^ cells/mL LCC9 cell in IMEM containing 5% CCS were transfected with control or PINK1 siRNA and were plated in 24-well tissue culture plates. On day 1 after plating, cells were treated with varying doses of fulvestrant (10 nM-1000 nM). On day 3, medium was aspirated and cells were stained with crystal violet. Cells were permeabilized using citrate buffer and absorbance was read at 660 nm using a plate reader.

To confirm the effect of treatments on autophagy and subcellular localization, western blot hybridization was used to measure LC3-I/LC3-II, p62, PINK1, parkin, and COXIV. Treated cell monolayers were solubilized in lysis buffer, protein was measured using a standard bicinchoninic acid assay, and proteins were size fractionated by polyacrylamide gel electrophoresis and transferred to nitrocellulose membranes. Non-specific binding was blocked by incubation with Tris-buffered saline containing 5% powdered milk and 1% Triton X-100. Membranes were incubated overnight at 4°C with primary antibodies, followed by incubation with polyclonal horseradish peroxidase (HRP)-conjugated secondary antibodies (1:2000) for 1 hour at room temperature. Immunoreactive products were visualized by chemiluminescence (SuperSignal Femto West, Pierce Biotechnology, Rockford, IL) and quantified by densitometry using the ImageJ digital densitometry software (http://rsbweb.nih.gov/ij/). Protein loading was visualized by incubation of stripped membranes with a monoclonal antibody to β-actin or β-tubulin (1:1000).

All data are presented as the mean ± standard error of the mean (SEM). Statistical differences were evaluated by one way analysis of variance (ANOVA) followed by Dunnett *post hoc* test. The criterion for statistical significance was set at p < 0.05 prior to initiation of the study.

## Results and discussion

**Figure 1:**
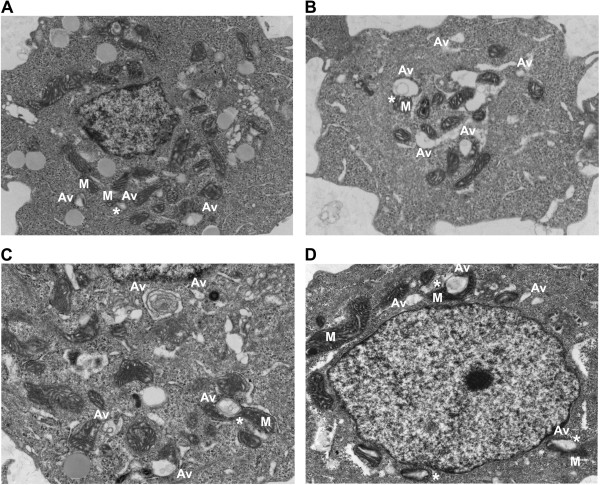
**Drug-induced autophagy in ER + breast cancer cells exhibit elevated autophagosome formation and indicate a direct contribution of the mitochondrial membrane in autophagosome membrane development.** Electron micrograph images of **A**. vehicle treated LCC9 breast cancer cells. **B**. LCC9 cells treated with 100 nM fulvestrant for 72 hours. **C**. LCC9 cells transfected with estrogen receptor-α shRNA. **D**. LCC9 cells treated with 10 μM Imatinib for 72 hours. M indicates mitochondria; Av indicates an autophagic vesicle; * indicates mitochondria-autophagosome interaction.

**Figure 2:**
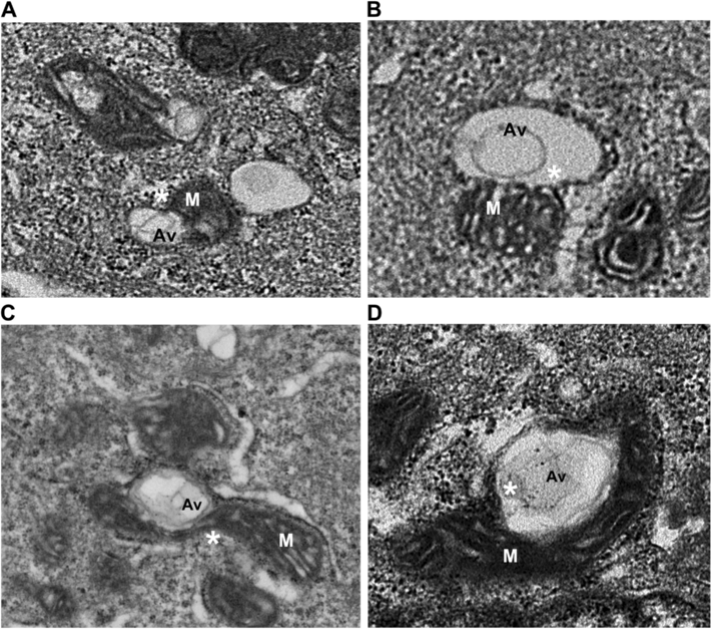
**Mitochondria directly donate membrane material to form autophagosomes.** Electron micrograph images of **A**. vehicle treated LCC9 breast cancer cells. **B**. LCC9 cells treated with 100 nM fulvestrant for 72 hours. **C**. LCC9 cells transfected with estrogen receptor-α shRNA. **D**. LCC9 cells treated with 10 μM Imatinib for 72 hours. M indicates mitochondria; Av indicates an autophagic vesicle; * indicates mitochondria-autophagosome interaction.

**Figure 3:**
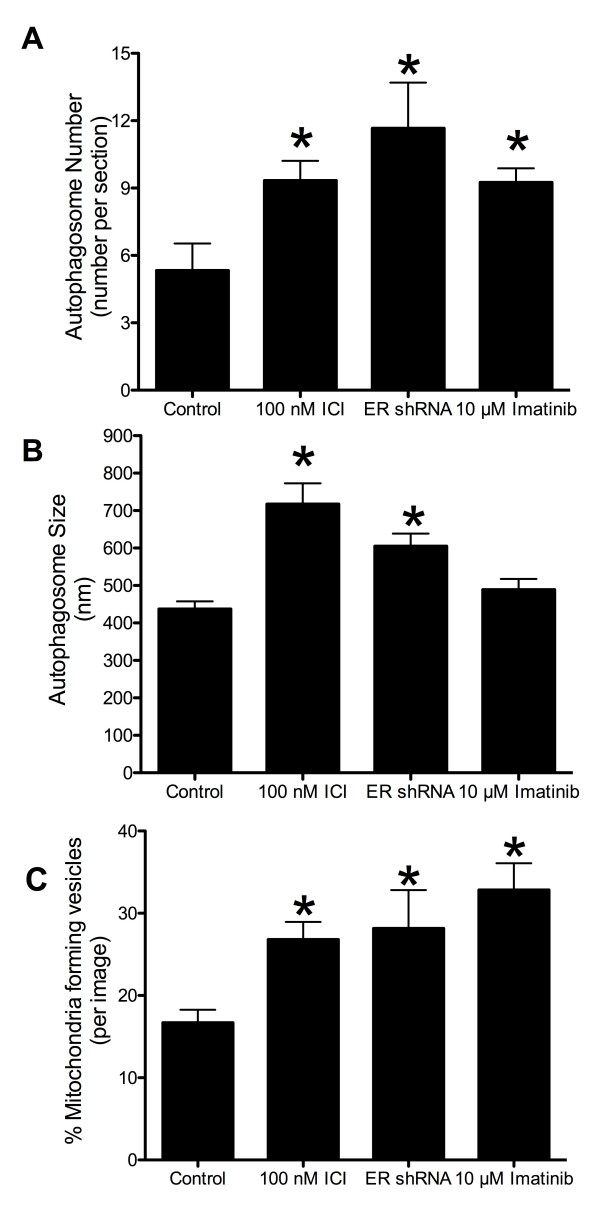
**Autophagosome number, size quantification, and percent mitochondria developing vesicles.** Autophagosomes were counted **(A)** and measured **(B)** using Image J software from electron microscopy images of LCC9 breast cancer cells. n = 3-5, *p < 0.05. **C**. Mitochondria were counted and graphed as percent of mitochondria forming vesicles. n = 5-7.

Autophagy is often increased in response to stress, starvation, and drug treatment [12]. Antiestrogens (tamoxifen (TAM) and Fulvestrant (ICI)) induce autophagy in ERα expressing human breast cancer cells [12-14]. This autophagy induction is associated with cell survival, suggesting that it is a major determinant of resistance to these drugs [15,16]. Using the LCC9 (ER+, estrogen independent, ICI resistant, TAM cross-resistant) [17] and MCF7 (ER+, estrogen dependent, ICI and TAM sensitive) breast cancer cell line, electron microscopy was used to investigate the effect of ER knockdown and treatment with antiestrogens and other autophagy-inducing drugs on autophagosome formation. Figure 1A shows that LCC9 vehicle treated (control) cells exhibit a high level of basal autophagy as indicated by the presence of autophagosomes marked Av (autophagic vacuole). Treatment with ICI increased the formation of autophagosomes (Figure 1B), as did ER knockdown that mimics the effects of ICI on ER expression (Figure 1C). Imatinib mesylate (Gleevec®), a c-abl inhibitor previously shown to induce autophagy in chronic myeloid leukemia cells [18], was also used to stimulate further autophagosome formation (Figure 1D). Higher magnification EM images show that mitochondria directly contribute their membrane material to form autophagosomes (Figure 2). In all electron microscopy images viewed in this study, we found at least one example of mitochondrial membranes forming contiguous structures with the membranes of developing autophagosomes (as indicated by *). Quantification of autophagosome number and size are shown in Figure 3. All treatments significantly increased autophagosome number, while antiestrogen therapy and ER shRNA treatments increased autophagosome size. The percentage of mitochondria forming these continuous vesicle-like structures was also determined (Figure 3C).

**Figure 4:**
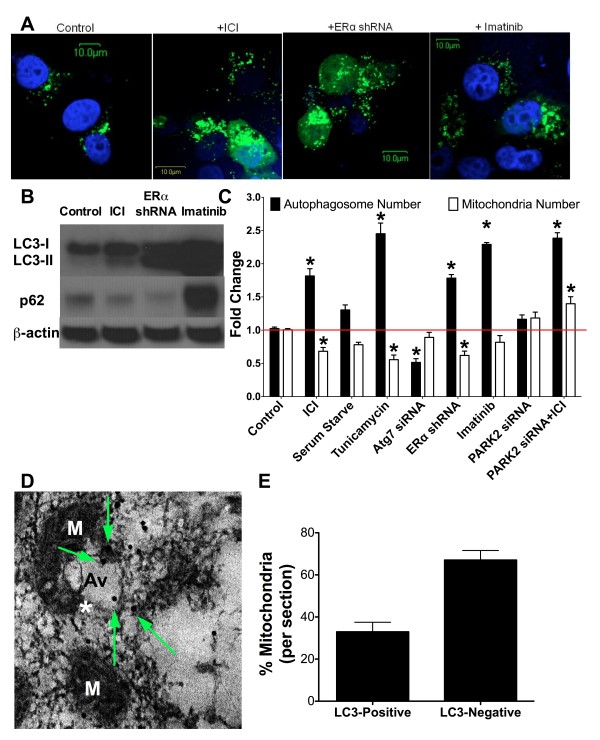
**Vesicles forming from mitochondria are autophagosomes. A**. LCC9 cells were transfected with GFP-LC3, treated with 0.1% ethanol vehicle, 500 nM ICI, ERα shRNA, or 10 μM Imatinib for 24 hours and counterstained with DAPI. Confocal microscopy was used to determine LC3-positive puncta formation. **B**. LCC9 cells were treated with 0.1% ethanol vehicle, 100 nM ICI, ERα shRNA, or 10 μM Imatinib for 72 hours and protein isolated. Western blot hybridization was used to determine LC3-I/LC3-II and p62 levels. **C.** LCC9 cells were treated with vehicle, 500 nM ICI, serum starvation, 2 ng/mL tunicamycin, 10 μM Imatinib, or transfected with ATG7 siRNA, PARK2 (parkin) siRNA, or ERα shRNA for 24 hours. Autophagosome (modified monodansylcadaverine) and mitochondria (MitoTracker-GFP) fold change was determined by flow cytometry. **D**. LC3-immunogold EM of LCC9 cells. M indicates mitochondria; Av indicates an autophagic vesicle; * indicates mitochondria-autophagosome interaction; arrows indicate LC3-immunogold particles. **E**. Mitochondria were counted and represented as percent of mitochondria either labeled with LC3-immunogold or unlabeled. n = 3-4, *p < 0.05.

To confirm induction of autophagy using these experimental conditions, LCC9 cells were transfected with GFP-LC3 and treated either with vehicle (control), 500 nM ICI, ERα shRNA, or 10 μM Imatinib for 24 hours. Confocal microscopy showed that antiestrogen therapy, knockdown of ERα, and Imatinib treatment each induced LC3-positive puncta formation, demonstrating that these conditions stimulate the initiation of autophagy (Figure 4A). Furthermore, western blot hybridization on protein lysates from LCC9 cells treated with vehicle control, 100 nM ICI, transfected with ER shRNA, or 10 μM Imatinib were used to confirm treatment effects on LC3 and p62 levels (Figure 4B). All treatments increased LC3-II formation in LCC9 cells, indicating that ICI, Imatinib, and ER knockdown increased autophagosome formation. ER knockdown and ICI treatment decreased p62 levels showing that these drugs increased autophagic flux. However treatment of LCC9 cells with Imatinib increased p62, suggesting that the c-abl inhibitor blocked autophagic flux by preventing lysosome degradation of autophagosomes. The effect of imatinib on autophagy and antiestrogen resistance will be further explored in future work. LCC9 cells were treated either with vehicle (control), or with serum starvation, 500 nM ICI, 2 ng/mL tunicamycin (as a positive control), ATG7 siRNA (as a negative control), ERα shRNA, or 10 μM Imatinib for 24 hours to increase autophagy. Prior to cell harvesting, cells were incubated with lentiviral MitoTracker-GFP. Cells were collected and treated with a modified monodansylcadaverine and sorted by flow cytometry to quantify autophagosome and mitochondria number (Figure 4C). Serum starvation, ICI, tunicamycin, ERα shRNA, or Imatinib treatment resulted in increased autophagosome formation. Serum starvation, ICI, tunicamycin, and ERα shRNA decreased overall mitochondrial content when compared with vehicle treated control. Imatinib had no significant effect on mitochondrial content; consistent with the maintenance of p62 levels indicating that autophagic flux is likely inhibited by drug treatment.

**Figure 5:**
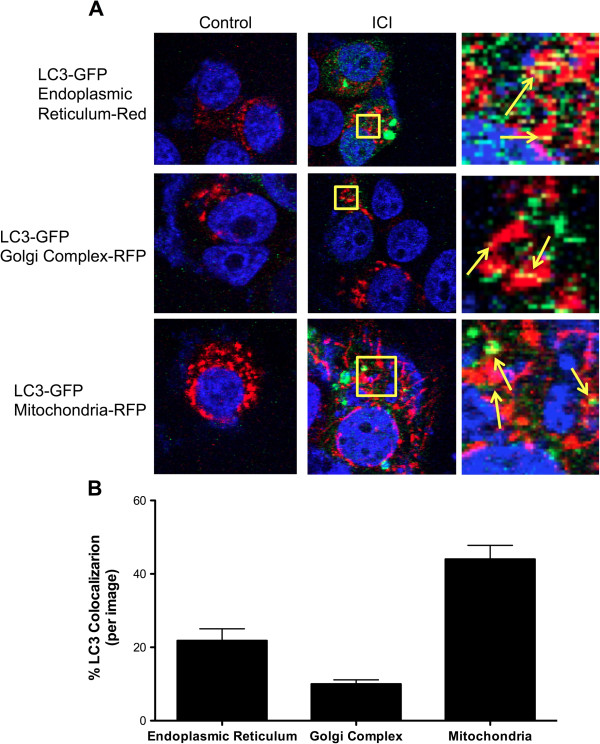
**Co-localization of autophagosomes and cellular organelles. A**. LCC9 cells were transfected with GFP-LC3 for 24 hours. Cells were incubated with either endoplasmic reticulum dye, Golgi complex-RFP, or MitoTracker-RFP and treated with 500 nM ICI to induce autophagy. Confocal microscopy was used to determine the co-localization of LC3 puncta with different cellular organelles. **B**. The percent of LC3-positive autophagosome co-localizing with different cellular structures was determined. n = 5-6.

Transfection of LCC9 cells with ATG7 siRNA to inhibit autophagy reduced basal autophagosome formation with no significant change in mitochondrial flux. Inhibition of mitophagy, via parkin knockdown, also inhibited mitochondrial flux with no effect on autophagosome formation. These data indicate a reciprocal relationship between autophagy and mitochondria, suggesting either that mitochondria are the cellular content of autophagosomes (mitophagy) and/or that mitochondria are being used as the “raw material” to form autophagosomes as we observe in Figure 2. Furthermore, co-localization of GFP-LC3 with Mitotracker-RFP, GolgiTracker-RFP, or EndoTracker (endoplasmic reticulum dye) was determined by confocal microscopy (Figure 5). We show that LC3 predominately localizes with the mitochondria. Moreover, images from LCC9 cells incubated with LC3-immunogold and studied by electron microscopy show that LC3 is localized to vesicles forming from mitochondria (Figure 4D), supporting the interpretation that the vesicles developing from mitochondria are autophagosomes. Quantification of the LC3-immunogold EM staining shows that the percentage of mitochondria per section that stain positive for LC3 is approximately 35% (Figure 4E). The percentage of mitochondria forming vesicles (Figure 3C) and the percentage of mitochondria stained positive for LC3 (Figure 4E) are similar, further supporting the likelihood that vesicles forming from mitochondria are autophagosomes. Data obtained by confocal microscopy confirm that these treatments induce autophagy, the flow cytometry data reflects both autophagosome and mitochondria flux, and the EM images show that mitochondrial membranes contribute to the formation of a membrane encapsulated autophagosomal-like vesicle, most likely reflecting the recycling of damaged or unnecessary mitochondria to form autophagosomes.

**Figure 6:**
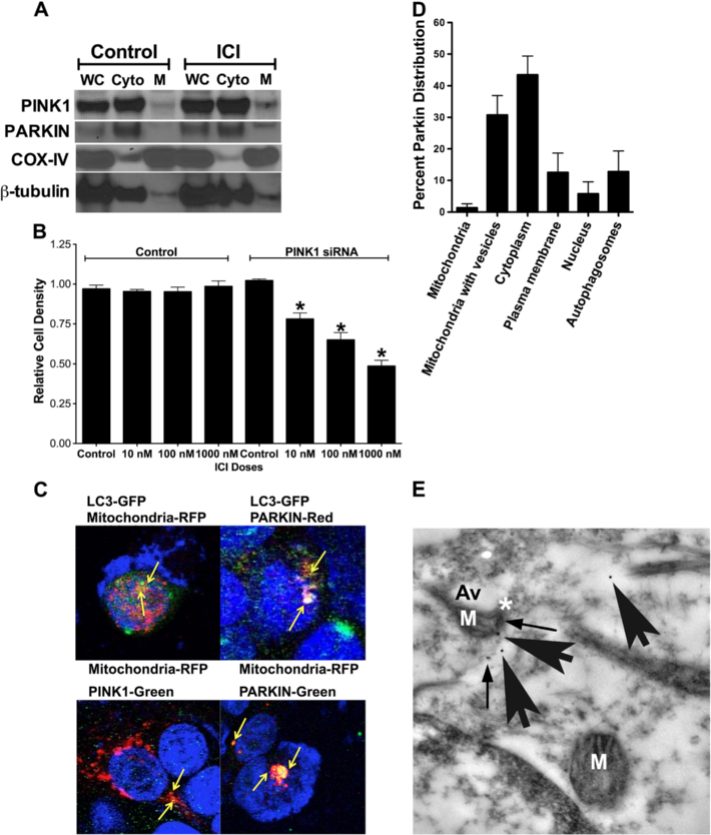
**Autophagosomes forming from mitochondria is a novel form of parkin-associated mitophagy. A**. Distribution of parkin and PINK1 in mitochondrial and cytoplasmic fractions of control and fulvestrant treated LCC9 cells as determined by western blot hybridization. **B**. Effect of mitophagy inhibition by PINK1 knockdown on antiestrogen sensitivity in LCC9 cells. **C**. Confocal microscopy of PINK1, parkin, LC3, and mitochondria in LCC9 cells treated with 100 nM ICI for 72 hours. **D**. Quantification of parkin-immunogold EM parkin distribution within LCC9 cells. n = 5. **E**. EM image of parkin-immunogold stained LCC9 cells. Mitochondria forming vesicles stain positive for parkin. M indicates mitochondria; Av indicates an autophagic vesicle; * indicates mitochondria-autophagosome interaction; arrows indicate parkin-immunogold particles.

Lastly, we investigated whether the mitochondria-forming autophagosomes may be a form of mitophagy. LCC9 cells were treated with vehicle control or 100 nM ICI for 72 hours. Mitochondrial or cytoplasmic protein fractions were collected and western blot hybridization performed to determine PINK1, parkin, COX-IV (mitochondrial control), or β-tubulin (cytoplasmic control). Treatment with ICI increased both PINK1 and parkin localization to the mitochondria (Figure 6A). Moreover, inhibition of mitophagy through PINK1 knockdown resensitized LCC9 cells to antiestrogen therapy, suggesting a dependence of LCC9 cells on functional mitophagy to maintain an antiestrogen resistant phenotype (Figure 6B). The antiestrogen resistant LCC9 human breast cancer cells exhibit an elevated level of endogenous parkin expression when compared with their endocrine sensitive parental cell line (data not shown), further supporting an important role of mitophagy in antiestrogen responsiveness. Additional studies into the mechanistic contribution of mitophagy to antiestrogen resistance are ongoing.

**Figure 7:**
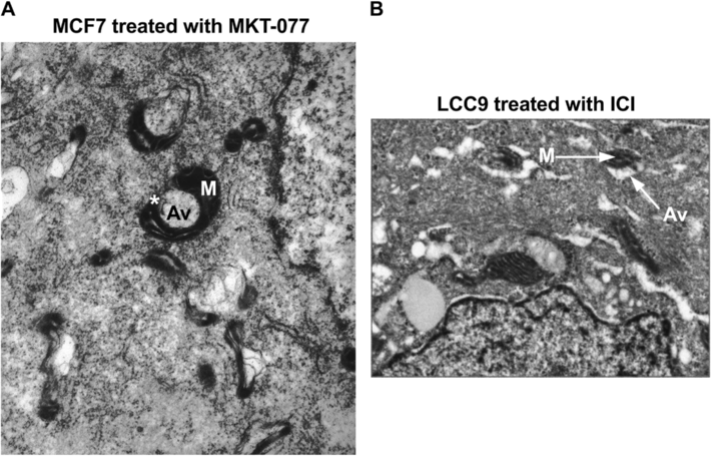
**Mitophagy in human breast cancer cells. A**. EM image of MCF7 cells treated with MKT-077, a cationic drug that concentrates in the mitochondria and inhibits glucose-regulated protein 75. Image indicates that mitochondria forming autophagosomes occur in other cell lines. **B**. EM image of LCC9 cells treated with ICI. Image shows an example of classical mitophagy where a mitochondria is engulfed by an autophagosome.

Confocal microscopy was performed on LCC9 cells treated with 100 nM ICI and either transfected with GFP-LC3 or incubated with a PINK1 antibody, parkin antibody, or mitotracker-RFP. As shown in Figure 6C when mitophagy is stimulated by ICI treatment, mitochondria localize with LC3, PINK1, and parkin. Moreover, LC3 also co-localizes with parkin, suggesting that mitochondria labeled with parkin are then either used to form autophagosomes or are engulfed by the forming autophagosomes. EM images suggest that both processes occur in ICI treated LCC9 cells; Figure 2 shows autophagosomes forming from mitochondria membranes, while Figure 7B shows an example of classical mitophagy where a mitochondria is localized inside a formed autophagosome. LCC9 cells were incubated with parkin-immunogold, and subsequent electron microscopy showed that parkin localized to mitochondria forming autophagosomes (Figure 6D). Thus, autophagosomes developing from mitochondria appear to represent a novel mechanism of mitophagy. Cellular parkin distribution is shown in Figure 6E, with parkin predominately localized within the cytoplasm and at mitochondria forming autophagosomes.

Autophagy is thought to occur naturally in most cells, and breast cancer cells often exhibit increased autophagy when compared with immortalized normal breast epithelial cells. Antiestrogen resistant breast cancer cells exhibit a further increase in autophagy when compared with their therapy sensitive counterparts [15,19,20]. We cannot exclude the possibility that these higher levels of autophagy in cancer cells result in the use of cellular materials or processes not commonly used in normal cells. Nonetheless, the use of preexisting target organelle membranes is an energy efficient process compared with *de novo* biosynthesis of a new double membrane, particularly if the membrane is at least partly obtained from the organelle being targeted for later degradation in the mature autolysosome. Moreover, we show that the process of mitochondrial-mediated autophagosome formation also occurs in MCF7 cells (ER+, antiestrogen sensitive breast cancer cells), implying that this phenomenon occurs more broadly than in just the LCC9 variant (Figure 7A). Since autophagy clearly plays an important role in breast cancer progression and therapeutic responsiveness [12,21,22], understanding how autophagy occurs may improve our ability to efficiently target this prosurvival pathway.

In conclusion, we show the first physical evidence, by electron microscopy, that mitochondria can supply membrane material during the creation of autophagosomes. We demonstrate that this occurs not only during serum starvation [8], but also during both basal (in the presence of serum and vehicle) and drug-induced autophagy. We go further to demonstrate that the autophagosomes developing from mitochondria may represent a novel mechanism of parkin-associated mitophagy, where mitochondrial membrane material can be contributed to formation of the developing autophagosome, rather than the autophagosome forming around parkin-labeled mitochondria. While we did not find similar early structures for autophagosomes incorporating other subcellular organelles, the data imply that the autophagic removal of Golgi/secretory vacuoles (crinophagy), endoplasmic reticulum (reticulophagy), and other organelles may also proceed with the contribution of target organelle membrane to formation of the membranes of the subsequent autophagosomes.

## Abbreviations

ANOVA: Analysis of variance; ATG5: Autophagy related gene 5; ATG7: Autophagy related gene 7; CCS: Charcoal stripped calf serum; DAPI: 4',6-diamidino-2-phenylindole; EM: Electron microscopy; ER: Estrogen receptor; GFP: Green fluorescent protein; ICI: Faslodex, fulvestrant, ICI 182,780; LC3: Microtubule-associated protein light chain 3; PARK2: Parkin; RFP: Red fluorescent protein; SEM: Standard error of the mean; TAM: Tamoxifen.

## Competing interests

Authors have no competing interests to declare.

## Authors’ contributions

KLC performed experiments, analyzed data, and wrote the manuscript. DSP, MA, and PAGC performed experiments and edited the manuscript. DDR and RC wrote and edited the manuscript. All authors read and approved the final manuscript.
